# Synergistic Enhancement of Hardness and Toughness in WC-Co Cemented Carbides Reinforced with (TiZrHfNbTa) (C, N) High-Entropy Carbonitride

**DOI:** 10.3390/ma19040731

**Published:** 2026-02-13

**Authors:** Zhenhao Shen, Shuanglong Zhao, Huan Liang, Guolong Yu, Yuting Zhang, Qiang Chen, Qiuyue Chen, Sergio González, Yuntao Xi, Xiaoyong Zhang, Hui Wang

**Affiliations:** 1 School of Materials Science and Engineering, Xi’an Shiyou University, Xi’an 710065, China; 13361580919@163.com; 2 State Key Laboratory for Advanced Metals and Materials, University of Science & Technology Beijing, Beijing 100083, China; zhaoshuanglong0105@163.com (S.Z.); lianghuan9342@163.com (H.L.); 15732582639@163.com (G.Y.); 15907373071@163.com (Y.Z.); 18800100448@163.com (Q.C.); 19196890301@163.com (Q.C.); 3 Department of Materials Science and Engineering, Universidad Carlos III de Madrid, Avda. Universidad 30, Leganes, 28911 Madrid, Spain; sergiogs10@gmx.co.uk

**Keywords:** WC-Co cemented carbides, high-entropy carbonitride, hardness–toughness synergy

## Abstract

The simultaneous enhancement of hardness and toughness in WC-Co cemented carbides remains a critical and persistent challenge for advanced cutting-tool applications, where conventional materials often suffer from inherent property trade-offs. In this study, a novel composite ceramic material—WC-(TiZrHfNbTa) (C, N) high-entropy carbonitride (HECN)-Co composite—was successfully fabricated via dry ball milling and spark plasma sintering (SPS) at 1300 °C following 90 h of ball milling. By incorporating varying amounts of HECN (0–15%, mass fraction, same below), the microstructure and mechanical properties of the composites were systematically tailored. The results demonstrate that the addition of HECN effectively refines the WC grains and increases the material density, leading to a pronounced improvement in hardness. Notably, the composite with 10% HECN (WC-10%HECN-9Co) exhibits an optimal balance of hardness and fracture toughness, achieving a Vickers hardness of 2375 ± 25 HV30 and a fracture toughness of 12.9 ± 1.1 MPa·m^1/2^. In contrast, excessive HECN addition (15 wt.%) induces excessive grain refinement, which significantly impairs toughness. Our study demonstrates that the introduction of (TiZrHfNbTa) (C, N) HECN as a reinforcing phase offers a viable and effective strategy for designing cemented carbides with an exceptional hardness–toughness synergy, showing great promise for demanding cutting applications such as high-speed machining and the processing of hard-to-cut materials.

## 1. Introduction

WC-Co cemented carbide (tungsten-carbide–cobalt-based cemented carbide), as a widely employed matrix material for cutting tools, possesses a combination of attractive properties including high hardness, substantial strength, excellent wear resistance, and notable corrosion resistance. However, with the continuous advancement of industrial technologies—such as the pursuit of higher transportation efficiency in the automotive and railway sectors, the development of ultra-deep well drilling in petroleum extraction, and the exploration of outer space in aerospace applications—traditional WC-Co cemented carbide is increasingly inadequate to meet the more stringent service requirements across various manufacturing fields. These limitations arise from inherent performance trade-offs, which have motivated extensive research efforts to overcome such constraints. In particular, achieving a simultaneous enhancement of both high hardness and high fracture toughness in WC-Co cemented carbide has long represented a critical technical challenge. Consequently, resolving this property trade-off has emerged recently as a key research focus [1,2,3,4,5].

The comprehensive mechanical properties of WC-Co cemented carbide can be enhanced through the addition of appropriate grain growth inhibitors (GGIs), such as VC, Cr_3_C_2_, TaC and TiC, etc. [6]. However, the incorporation of a single GGI often leads to a conflict between grain refinement and the weakening of interfacial bonding [7], making it difficult to achieve simultaneous improvements in both hardness and toughness. Among these inhibitors, VC is recognized as the most effective in suppressing abnormal grain growth of WC. Nevertheless, VC tends to form (W, V) C_x_ complexes at the WC/Co interface, which can continuously develop into a nanoscale film covering the WC surface. This film disrupts the direct bonding between WC and the Co binder, reduces intergranular cohesion, and consequently leads to a significant decline in fracture toughness. The tendency of a single inhibitor to form such complexes arises from its composition containing only one metallic element, which promotes efficient segregation at the WC/Co interface. Moreover, the resulting complexes exhibit low lattice mismatch and low formation energy relative to WC, favoring their spontaneous and thermodynamically stable growth [8,9,10,11]. Therefore, further in-depth research remains necessary to design modification strategies for cemented carbides that can effectively balance high hardness with high fracture toughness.

High-entropy carbonitrides (HECNs), which benefit from the high mixing entropy effect of their multi-principal-element systems and the associated unique microstructural, electronic, and deformation characteristics, inherently exhibit a promising combination of high hardness and fracture toughness [12]. Moreover, the competitive diffusion and adsorption among their multiple constituent elements lead to a more homogeneous distribution of elements at interfaces. The introduction of multiple dopants increases the formation energy of secondary phases, intensifies lattice distortion, disrupts interfacial coherence with WC, and consequently suppresses the formation and stabilization of brittle interfacial complexes [13,14,15,16,17]. These attributes make HECNs a high-potential novel reinforcing phase for WC-Co-based cemented carbides. For instance, the WC–(TiTaMoVCr) (C, N)–9Co composite prepared by Ma et al. via SPS achieved a Vickers hardness of 1439 kg·mm^−2^ and a fracture toughness of 12.19 MPa·m^1/2^ [18]. Similarly, Liu et al. through vacuum liquid-phase sintering reported that a WC–(TiWMoVNb) (C, N)–10Co composite exhibited a Vickers hardness of 21.58 ± 0.36 GPa and a fracture toughness of 12.27 ± 1.17 MPa·m^1/2^ [19]. These results demonstrate that incorporating HECNs as a reinforcing phase into WC-Co cemented carbides can significantly enhance alloy hardness while maintaining relatively high toughness.

Despite the growing research interest in HECN powders for simultaneously enhancing the hardness and toughness of cemented carbides, systematic literature reports on this topic remain scarce. In this case, (TiZrHfNbTa) (C, N) HECN powders were synthesized via dry ball milling and subsequently employed to fabricate WC-10% (TiZrHfNbTa) (C, N)-9Co cemented carbide composites via SPS. The sintered composites exhibited a Vickers hardness of 2375 ± 25 HV30 and a fracture toughness of 12.9 ± 1.1 MPa·m^1/2^, and their performance is significantly superior to that of similar materials reported to date. This improved combination of properties suggests promising potential for applications in high-performance cutting tools, particularly under demanding conditions such as high-speed machining and the machining of hard-to-cut materials.

## 2. Experimental

### 2.1. Synthesis of WC-(TiZrHfNbTa) (C, N)-Co High-Entropy Carbonitride Composite

**Figure 1:**
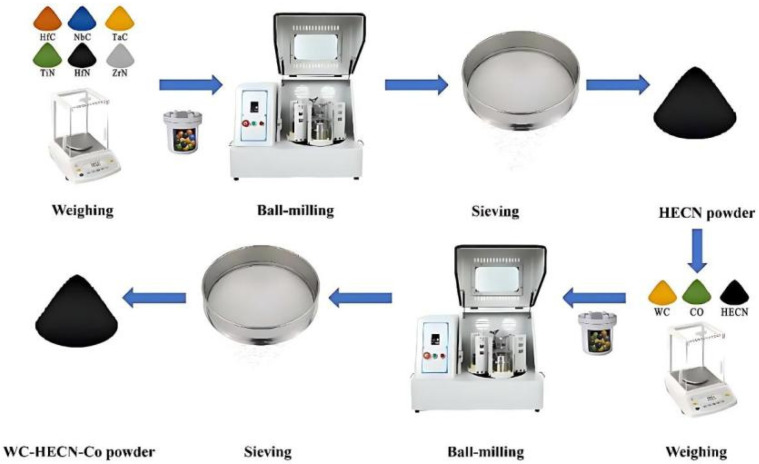
Schematic synthesis process for WC-HECN-Co composite powders. All procedures, except ball milling, were performed in a glove box.

The starting materials consisted of commercially available HfC, NbC, TaC, TiN, ZrN, HfN, WC, and Co powders (purity: 99.5%; average particle size: 1–3 μm), which were purchased from Beijing Licheng Chuangxin Metal Materials Technology Co., Ltd., Beijing China. The (TiZrHfNbTa) (C, N) HECN powders were prepared as follows: HfC, NbC, TaC, TiN, ZrN, and HfN powders were weighed according to the stoichiometric ratio of the target HECN and mixed in a stainless-steel ball-mill jar. The (TiZrHfNbTa) (C, N) HECN powders were synthesized after the powders were ball-milled at 300 rpm for 10, 30, 50, 70 or 90 h, respectively, with a ball-to-powder weight ratio of 5:1, where stainless-steel balls and stearic acid were used as grinding media and a process-control agent, respectively. Subsequently, WC powders, 9Co powders, and the synthesized HECN powders (with different contents of 5.0, 10.0, and 15.0%, respectively) were blended and further milled under the same conditions for 10 h to obtain homogeneous WC-HECN-Co composite powders. The overall powder-preparation procedure is illustrated in Figure 1**.** Bulk specimens with a diameter of 10 mm were consolidated by spark plasma sintering (SPS-211H, Fuji Electronic Industrial Co., Ltd., Kawagoe, Saitama, Japan), which was performed at 1300 °C under a uniaxial pressure of 50 MPa, with a heating rate of 100 °C min^−1^ and a holding time of 5 min.

### 2.2. Characterization

The microstructure of the sintered samples was characterized using X-ray diffraction (XRD; DMAX-RB-12 KW, Rigaku Corporation, Cu-Kα radiation, Tokyo, Japan). XRD scans were performed over a 2θ range of 30–65° at a scan rate of 5°/min for powder samples and 20~80° at 10° min^−1^ for bulk samples. Microstructural morphology and elemental analysis were examined using scanning electron microscopy (SEM; Zeiss Supra 55, Oberkochen, Germany) equipped with energy-dispersive X-ray spectroscopy (EDS). Electron backscatter diffraction (EBSD) was performed using an Oxford Instruments Symmetry S2 detector (Oxford Instruments, Abingdon, Oxfordshire, UK) on a Thermo Scientific Apreo 2S SEM (Thermo Fisher Scientific, Waltham, MA, USA) at 20 kV and 12 nA, with a scan speed of ~1500 pts/s and a step size of 0.02 μm, yielding data with an average CI of 0.45 and an indexing rate > 97%. The actual density of the samples was determined by the Archimedes method. Vickers hardness was measured under a load of 30 kgf (HV30) using a digital microhardness tester (7MHVS-50A, Laizhou Huaxing, Laizhou, China), with a dwell time of 15 s. The fracture toughness, *K*_I*C*_, can be obtained by the following Equation (1) [20]:

(1) $$K_{IC}=0.15\sqrt{\frac{\mathrm{HV}30}{\sum_{i=1}^{4}l_{i}}}$$

where *HV*30 refers to the Vickers hardness value and $l_{i}$ denotes the total length of the crack, in this case, the total length of the four corner cracks of the square-based pyramid-shaped indent (mm).

## 3. Results and Discussion

### 3.1. Powder Characterization

**Figure 2:**
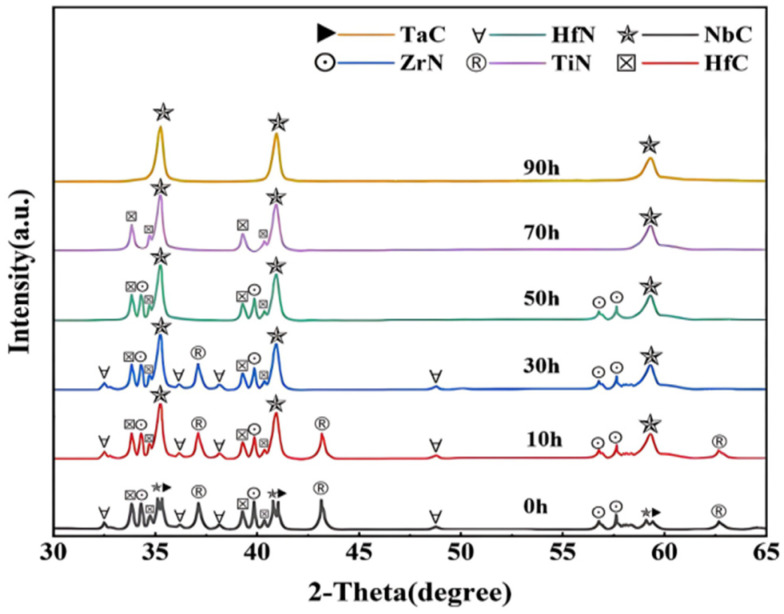
XRD patterns of HECN powders under different ball-milling durations.

**Figure 3:**
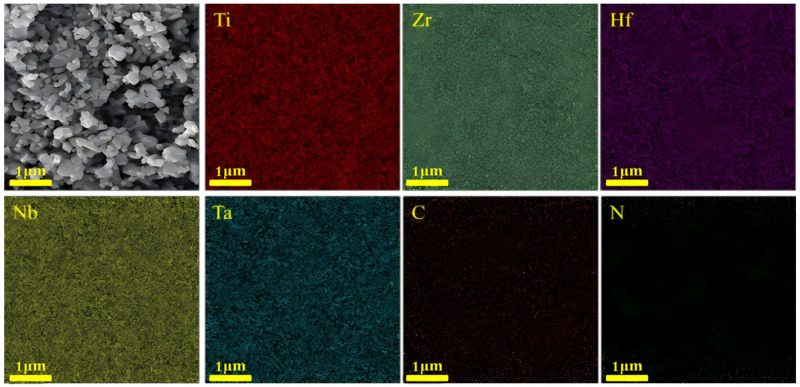
The SEM image and mapping of HECN powders after 90 h dry ball milling.

The XRD patterns of the HECN powders milled for different durations are displayed in Figure 2, indicating that the mechanical alloying process, especially ball-milling time, has a significant impact on the structure. With ball-milling durations of 10 h, 30 h, 50 h, and 70 h, the constituent elements, TaC, TiN, HfN, and ZrN, due to their diffusion kinetics, gradually dissolve into the NbC lattice to form a solid solution, respectively, and finally the matrix structure is dominated by NbC. The SEM-EDS elemental mapping results of the HECN powders after 90 h of ball milling are presented in Figure 3, which demonstrate a uniform spatial distribution of all constituent elements, with no detectable elemental enrichment or segregation, and verify that a homogenized high-entropy solid solution was successfully achieved through the applied mechanical alloying process.

### 3.2. Phase Composition

**Figure 4:**
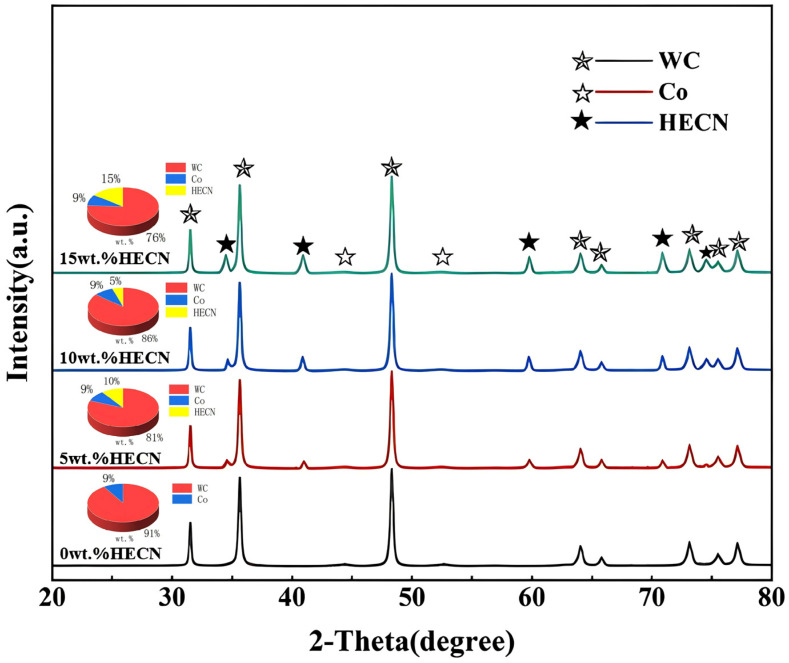
XRD patterns of WC-Co-based cemented carbides with different HECN additions subjected to SPS.

Figure 4 presents the X-ray diffraction (XRD) patterns of the sintered WC-(TiZrHfNbTa) (C, N)-Co high-entropy carbonitride composite with varying additions of HECN powder. The patterns confirm that all composites sintered at 1300 °C consist solely of WC, Co, and HECN phases, with no detectable diffraction peaks from impurity phases. As the HECN content increases, the intensity of the characteristic diffraction peaks for the HECN phase increases correspondingly. Conversely, due to the reduced relative mass fraction of WC in the composite, the diffraction peak intensities of the WC phase exhibit a systematic decrease. Owing to the presence of high-atomic-number elements (Ta, Hf) in the HECN, which possess significantly higher atomic scattering factors than Co, the HECN phase yields detectable diffraction peaks, whereas the diffraction signals of the Co phase remain extremely weak, even at the same low mass fraction. Quantitative phase analysis was conducted on the samples with different HECN contents, and the obtained results were consistent with the addition amounts designed in the experiment. A systematic shift of the WC diffraction peaks to higher angles with increasing HECN content is clearly observed in the detailed XRD analysis, indicating a reduction in interplanar spacing and the presence of compressive lattice strain within the WC phase. This peak shift was quantitatively assessed using two complementary methods: lattice parameter refinement via the Rietveld method and direct peak shift analysis. Rietveld refinement of the entire diffraction pattern confirmed a gradual contraction of the WC unit cell, with the lattice parameter ‘a’ decreasing from 2.906 Å (0 wt.% HECN) to 2.899 Å (15 wt.% HECN). Concurrently, analysis of the shift in the (101) peak yielded a consistent compressive strain of approximately 0.24%. This induced compressive stress field is attributed to the mismatch in the coefficients of thermal expansion (CTEs) between the WC matrix and the HECN particles, which constitutes a key factor contributing to the enhanced synergy of hardness and toughness in the composite.

**Figure 5:**
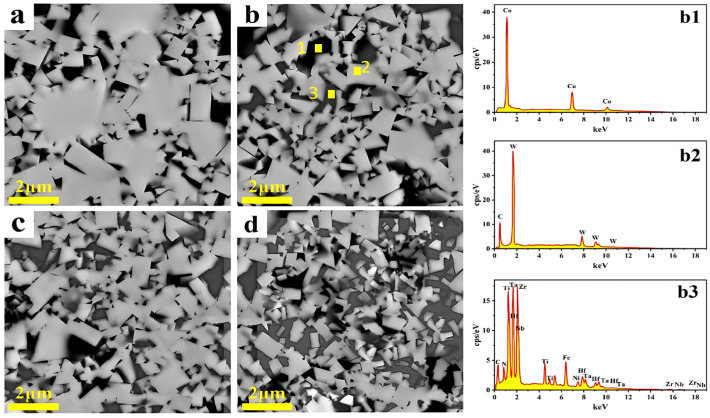
BSE images of WC-Co-based cemented carbides with different HECN additions, where (**a**) 0, (**b**) 5%, (**c**) 10%, (**d**) 15%; (**b1**–**b3**) correspond to the point scanning results of Points 1, 2, 3, respectively (for 5%).

The backscattered electron (BSE) images of the composite polished surfaces with different HECN contents are shown in Figure 5. The image contrast is determined by the average atomic number of each phase, a characteristic that enables preliminary phase identification: the light gray regions (with the highest average atomic number) correspond to the tungsten carbide (WC) phase (Z≈40), the dark gray regions to the (TiZrHfNbTa) (C, N) HECN phase (Z≈28), and the black regions to the Co binder phase (Z ≈ 27). According to the XRD results shown in Figure 4, only two sets of diffraction peaks are present in the WC-Co cemented carbide. With an increase in the HECN content, the diffraction peaks corresponding to HECN gradually intensify. As shown in Figure 5a, visual observation of the images also reveals that no dark gray phase is detected in the HECN-free WC-Co cemented carbide, where only light gray and black phases are present. In contrast, as the HECN content increases in Figure 5b–d, the area of the dark gray regions expands progressively. This observation further infers that the dark gray phase corresponds to the HECN phase. Moreover, the results of the point scan analysis of the WC-5HECN-9Co composite (Points 1, 2, and 3 in Figure 5b) verify the validity of the aforementioned phase analysis.

**Figure 6:**
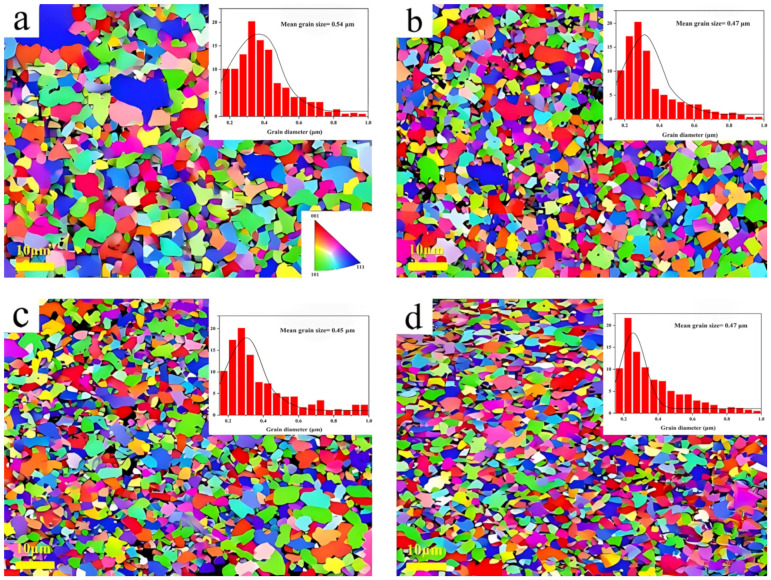
EBSD images and grain size statistics of WC-Co-based cemented carbides with different HECN additions, (**a**) 0, (**b**) 5%, (**c**) 10%, and (**d**) 15%.

The effect of HECN content on microstructure refinement is quantified in Figure 6**,** which shows EBSD maps and the corresponding grain size distributions. As the HECN content is gradually increased from 0 to 15%, a significant reduction in the average grain size of the WC-Co composite can be observed. When the mass fraction of HECN is increased to 15%, the average grain size decreases from 0.54 ± 0.05 μm to 0.39 ± 0.05 μm, and a substantial grain refinement is achieved at this point. HECN particles inhibit WC grain boundary migration via the grain boundary pinning effect, while reducing the dissolution and precipitation of WC in the cobalt phase, weakening the driving force for grain growth, and ultimately achieving significant grain refinement.

### 3.3. Mechanical Properties

**Figure 7:**
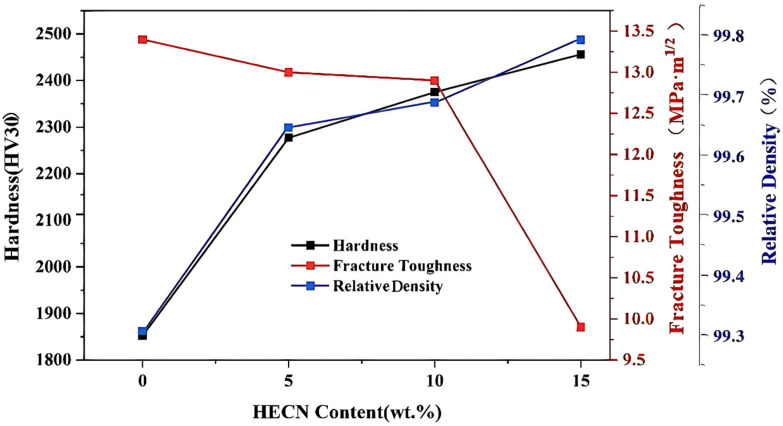
Relative density (herein refers to measured density to theoretical density ratio), hardness and fracture toughness of WC-Co-based cemented carbides with different HECN additions as a function of HECN content.

**Figure 8:**
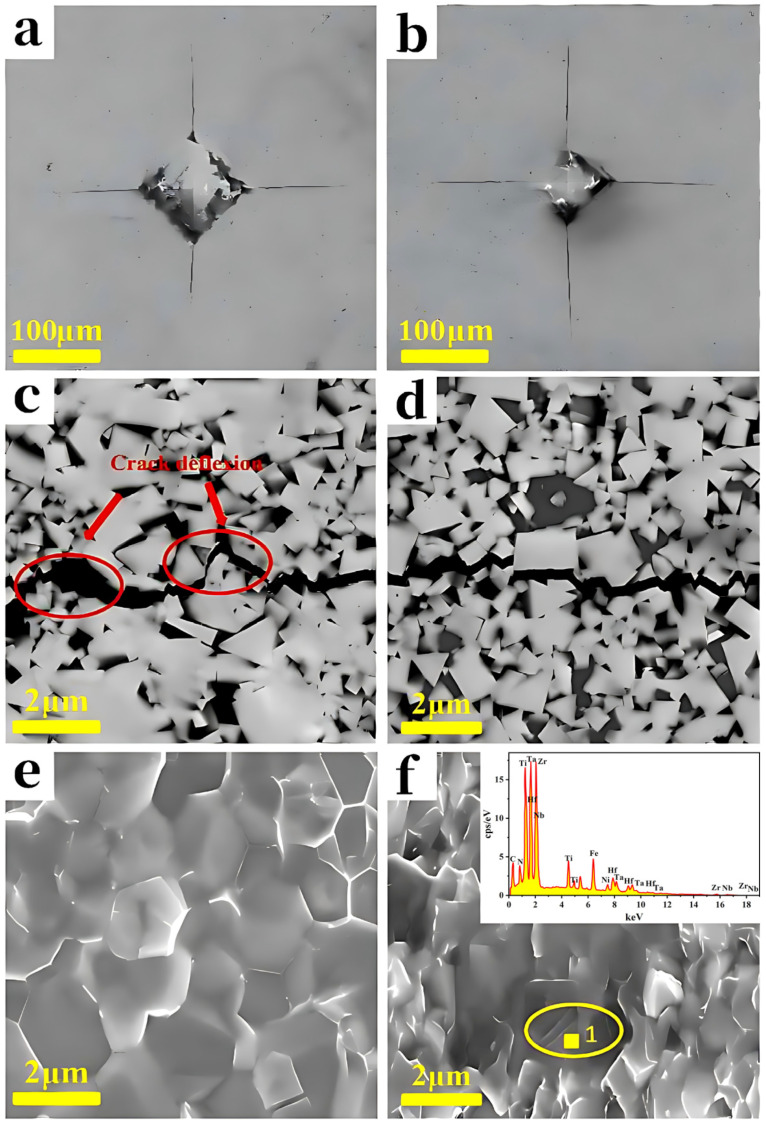
Indentation (**a**), Crack propagation paths (**c**) and fracture morphologies (**e**) of WC-9Co cemented carbides; Indentation (**b**), crack propagation paths (**d**) and fracture morphologies (**f**) of WC-10%HECN-9Co cemented carbides, as well as the point scanning results of Point 1 on the dissociation terrace (marked by the small yellow square).

The hardness, fracture toughness, and relative density (the detailed density-related data are presented in Table 1) of WC-(TiZrHfNbTa) (C, N)-Co high-entropy carbonitride composites with different additional HECN contents are shown in Figure 7. A positive correlation is observed between relative density and hardness. Both properties increase monotonically with higher HECN additions, showing a consistent trend across the composition range. The composite with 15HECN achieved the highest values, with a hardness of 2456 ± 25 HV30 and a relative density of 99.8 ± 0.1%. Based on the observations of indentations on WC-Co and WC-10%HECN-Co as shown in Figure 8a,b, the indentation area of the sample with 10% HECN addition decreased significantly compared to that of the sample without HECN. The increase in hardness can be attributed to two key microstructural factors. As established previously, the grain size is progressively refined with increasing HECN content and the hardness and grain size are characterized by the Hall–Petch relationship as below [21]:

(2) $$HV=HV_{0}+k_{HV}\cdot d^{-1/2}$$

where $HV_{0}$ is the hardness when the grain size is infinitely large (a constant); $k_{HV}$ is the grain refinement strengthening coefficient related to hardness. According to Equation (2), the grain refinement directly contributes to higher hardness. Second, the fine and uniform grain structure can significantly shorten the atomic diffusion distance during sintering and effectively improve the rate of mass transport and the efficiency of pore elimination, thereby laying a solid microstructural foundation for the full densification of the material. The resulting dense microstructure can optimize the load transfer efficiency among the WC phase, HECN phase and Co binder phase, reduce the formation of internal stress concentration sites, and further impede dislocation motion, ultimately leading to a remarkable enhancement in the overall hardness of the material.

In contrast, the fracture toughness of the composites decreased with increasing HECN content. For WC-Co cemented carbides, fracture toughness is primarily governed by the Co binder content and the WC grain size. Under constant Co content and sintering conditions, a larger WC grain size typically enhances toughness [22]. This is because WC crystals possess only four independent slip systems. As the grain size increases, crack deflection and crack branching become more pronounced, thereby extending the fracture path and contributing to toughening [23,24]. The observed decrease in fracture toughness with higher HECN additions can therefore be attributed to the progressive grain refinement confirmed earlier. Notably, the decline in fracture toughness was marginal at HECN contents up to 10%, decreasing only from 13.4 ± 1.1 MPa·m^1/2^ (free HECN) to 12.9 ± 1.1 MPa·m^1/2^ (10 HECN)—a reduction of merely 3.7%. However, a sharp drop to 9.9 ± 1.1 MPa·m^1/2^ occurred when the HECN content was increased to 15%, representing a substantial loss in toughness compared to the HECN-free baseline.

To elucidate the mechanisms underlying the marginal toughness reduction at 10% HECN and its dramatic decline at 15%, the crack propagation and fracture morphologies were examined. As displayed in Figure 8c,d, crack expanding paths in the composites contenting 0 and 10% HECN are compared. In the WC-9Co sample, the crack path is tortuous, dominated by deflection at coarse WC grain boundaries. In contrast, the crack path in the WC-10%HECN-9Co sample is relatively straight, reflecting the limited crack deflection capability due to grain refinement. The corresponding fracture surfaces are presented in Figure 8e,f. The WC-9Co sample fails predominantly via intergranular fracture. In the WC-10%HECN-9Co sample, a mixed fracture mode is observed, involving both intergranular fracture and transgranular cleavage through fine grains. Cleavage steps, a typical feature of transgranular fracture, are evident on the fracture surface (marked by yellow circles in Figure 8f). EDS point analysis of Point 1 in Figure 8f confirmed that these cleavage facets correspond to the HECN phase. These results indicate that, although grain refinement generally impairs toughness, the introduction of the HECN phase promotes a mixed intergranular–transgranular fracture mode. This mode consumes additional fracture energy, thereby partially mitigating the toughness degradation associated with grain refinement. However, for 15HECN, the grain size is refined to 0.39 ± 0.05 μm. At this scale, the excessive grain refinement severely limits crack path tortuosity. The beneficial effect of the altered fracture mode induced by HECN becomes insufficient to offset the pronounced toughness loss caused by the ultra-fine microstructure, ultimately leading to the sharp decline in fracture toughness.

## 4. Conclusions

We demonstrated that the effective use of a novel (TiZrHfNbTa) (C, N) HECN to tailor the microstructure and mechanical properties of WC-Co cemented carbides. Key outcomes are highlighted as follows:

1. A single-phase (TiZrHfNbTa) (C, N) HECN powder was synthesized via 90 h of dry ball milling. When incorporated into WC-Co composites and consolidated by spark plasma sintering at 1300 °C, the HECN addition effectively refined the WC grains, reducing the average size from 0.54 ± 0.05 μm to 0.39 ± 0.05 μm with 15% addition, without formation of impurities.
2. The composites exhibited a progressive increase in hardness and density with higher HECN content. Fracture toughness, however, showed a critical dependence on the HECN-induced microstructure: at ≤10% HECN, a mixed intergranular–transgranular fracture mode maintained high toughness (~12.9 ± 1.1 MPa·m^1/2^), while at 15%, excessive grain refinement severely limited crack deflection, causing a pronounced toughness drop to 9.9 ± 1.1 MPa·m^1/2^.
3. An optimal hardness–toughness synergy was achieved in the WC-10%HECN-9Co composite, which exhibited a Vickers hardness of 2375 ± 25 HV30 and a fracture toughness of 12.9 ± 1.1 MPa·m^1/2^. This superior balance of properties positions the developed composite as a promising candidate for advanced cutting-tool applications.

## Figures and Tables

**Table 1:** Density Data of the Prepared Samples.

Composition (wt.%)	Theoretical Density (g·cm^−3^)	Actual Density (g·cm^−3^)	Relative Density (%)
**WC-9Co**	14.75	14.65	99.32
**WC-5HECN-9Co**	14.99	14.93	99.64
**WC-10HECN-9Co**	15.24	15.19	99.67
**WC-15HECN-9Co**	15.49	15.46	99.79

## Data Availability

The original contributions presented in this study are included in the article. Further inquiries can be directed to the corresponding authors.
